# The receptive versus current risks of *Plasmodium falciparum* transmission in Northern Namibia: implications for elimination

**DOI:** 10.1186/1471-2334-13-184

**Published:** 2013-04-23

**Authors:** Abdisalan M Noor, Petrina Uusiku, Richard N Kamwi, Stark Katokele, Benson Ntomwa, Victor A Alegana, Robert W Snow

**Affiliations:** 1 Malaria Public Health Department, Kenya Medical Research Institute-Wellcome Trust-University of Oxford Collaborative Programme, P.O. Box 43640, Nairobi, 00100 GPO, Kenya; 2 Centre for Tropical Medicine, Nuffield Department of Clinical Medicine, University of Oxford, CCVTM, Oxford, OX 3 7LJ, UK; 3 Office of the Minister, Ministry of Health and Social Services, Private Bag 13198, Windhoek, The Republic of Namibia; 4 Directorate of Special Programmes, National Vector-borne Diseases Control Programme, Private Bag 13198, Windhoek, The Republic of Namibia

## Abstract

**Background:**

Countries aiming for malaria elimination need to define their malariogenic potential, of which measures of both receptive and current transmission are major components. As Namibia pursues malaria elimination, the importation risks due to cross-border human population movements with higher risk neighboring countries has been identified as a major challenge. Here we used historical and contemporary *Plasmodium falciparum* prevalence data for Namibia to estimate receptive and current levels of malaria risk in nine northern regions. We explore the potential of these risk maps to support decision-making for malaria elimination in Namibia.

**Methods:**

Age-corrected geocoded community *P. falciparum* rate *Pf*PR_2-10_ data from the period 1967–1992 (n = 3,260) and 2009 (n = 120) were modeled separately within a Bayesian model-based geostatistical (MBG) framework. A full Bayesian space-time MBG model was implemented using the 1967–1992 data to make predictions for every five years from 1969 to 1989. These maps were used to compute the maximum mean *Pf*PR_2-10_ at 5 x 5 km locations in the northern regions of Namibia to estimate receptivity. A separate spatial Bayesian MBG was fitted to the 2009 data to predict current risk of malaria at similar spatial resolution. Using a high-resolution population map for Namibia, population at risk by receptive and current endemicity by region and population adjusted *Pf*PR_2-10_ by health district were computed. Validations of predictions were undertaken separately for the historical and current risk models.

**Results:**

Highest receptive risks were observed in the northern regions of Caprivi, Kavango and Ohangwena along the border with Angola and Zambia. Relative to the receptive risks, over 90% of the 1.4 million people across the nine regions of northern Namibia appear to have transitioned to a lower endemic class by 2009. The biggest transition appeared to have occurred in areas of highest receptive risks. Of the 23 health districts, 12 had receptive PA*Pf*PR_2-10_ risks of 5% to 18% and accounted for 57% of the population in the north. Current PA*Pf*PR_2-10_ risks was largely <5% across the study area.

**Conclusions:**

The comparison of receptive and current malaria risks in the northern regions of Namibia show health districts that are most at risk of importation due to their proximity to the relatively higher transmission northern neighbouring countries, higher population and modeled receptivity. These health districts should be prioritized as the cross-border control initiatives are rolled out.

## Background

Namibia has declared ambitions to eliminate malaria by 2020 [1,2] and this is embodied in the country's vision for 2030 that aims to abolish diseases of poverty [3]. The feasibility of an elimination agenda requires an understanding of the political and economic sustainability, logistic and operational challenges and the biological basis of receptive and current levels of malaria transmission [4,5]. Namibia has demonstrated a huge political commitment [6], is a regional emerging economy [7] and has a long legacy of control operations [8-12]. Despite significant declines in clinical incidence, the largest challenge facing elimination of malaria in Namibia remains the perennial threat of imported infections within the country and across its border with Angola [2,13,14]. Although infection rates have declined in northern Namibia, the *Anopheles* vectors remain and asymptomatic infected individuals who travel to this region can contribute to resurgent malaria transmission. To quantify this importation risk requires an understanding of the malariogenic potential [15,16], of which the receptive risk, also known as the intrinsic transmission potential and current risk, are major components [17]. Here we combine previously modelled estimates of the changing *Plasmodium falciparum* malaria risk in Namibia between 1969 and 1989 [12] with modelled predictions of current, 2009, risk to define areas of probable high rebound risks in the event of importation of infections into northern Namibia.

## Methods

### Country context

**Figure 1:**
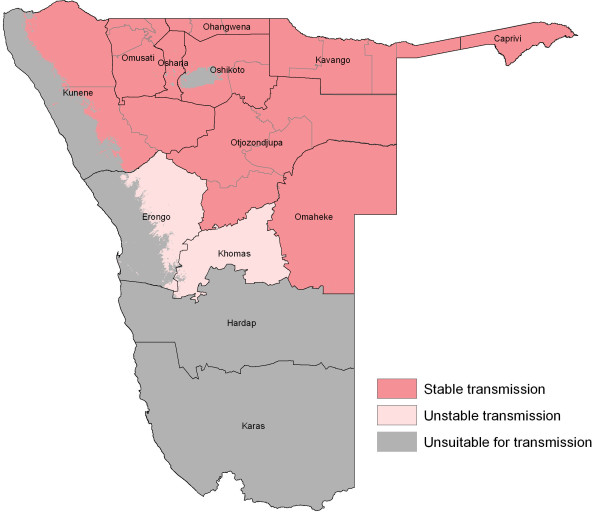
**Map of current first level administrative units (black line) and health districts (grey line) of Namibia showing the spatial limits of *P. falciparum *transmission.** Predictions of receptive and current *Pf*PR_2-10_ were restricted to the stable limits of transmission. Approaches used to constructing the spatial limits of *P. falciparum* transmission for Namibia are provided in [11].

Namibia was declared independent in March 1990 from South Africa which had ruled the country since 1919 [18]. It currently has a population of approximately 2.2 million people in an area of approximately 0.83 million km^2^[18,19] making it one of the most sparsely populated countries in the world. However 65% of the population live in 55% of the country's land mass that make up the nine regions of the north (Figure 1) which historically have been the most malarious [12].

The dominant vector of malaria in Namibia today is *Anopheles arabiensis.* The indoor resting vectors *An. gambiae s.s* and *An. funestus,* despite their historical role in transmission, have diminished following aggressive control activities, but continue to exist in some foci. *Plasmodium falciparum* represents the majority of all human malaria infections although cases of *P. malariae,* and *P. vivax* in Bushmanland, have been reported [7,19-21]. The main rainy season in Namibia runs from November to April and peaks in February to March but the total precipitation is extremely variable from year-to-year making transmission acutely seasonal and prone to epidemics [21-23].

Extreme aridity limits transmission along the Atlantic Coast, in the Namib Desert, parts of the Kalahari Desert in the South and the Etosha and other smaller saltpans (Figure 1). The southern regions to the Orange River on the border with South Africa have been defined as largely free of transmission or with rare occurrence of malaria cases and include the regions of Hardap, Karas and southern Omaheke [2,11,22,24]. However, some focal risks continue to exist in parts of Erongo, Khomas and the northern parts of Omaheke and these areas are best described as supporting unstable transmission [11,12].

Bi-annual indoor residual spraying (IRS) using dichlorodiphenyltrichloroethane (DDT) started in 1965 and later expanded to all malarious areas in the northern territories. Darachlor (chloroquine + pyrimethamine) was used as part of mass-drug administration to complement vector control activities [8-12]. Between 1966 and 1979, over 1.6 million kilograms of DDT was used to spray approximately 12.4 million housing structures and over 6.7 million tablets of Darachlor were distributed [12]. In 1980 Bendiocarb was introduced to replace DDT in urban areas. By the late 1980s intervention coverage began to decline, in part as a result of the war for independence, and combined with the failure of cholroquine for treatment, a series of epidemics followed [12].

Following the epidemic of 1990, the National Malaria Control Programme, later renamed the National Vector-borne Disease Control Programme was set up by the Ministry of Health and Social Services [21]. In 1995, a national malaria policy and strategy covering the period 1996–2001 was launched to improve the coverage of early diagnosis and treatment, targeted vector control and establishing epidemic early warning systems [21]. DDT continued to be the mainstay of IRS, however in 2005 Deltamethrin replaced Bendiocarb as a supporting residual insecticide [22]. Since 2001, IRS coverage has remained above 80% of the targeted households except in 2008 when the supplier delayed in procuring insecticides on time and coverage dropped to 38% [2]. Over the last 10 years, it is estimated that malaria case incidence and mortality in Namibia have been reduced by over 80% [2]. Consequently the southern parts of the regions of Kunene and Omaheke and all of Erongo, Hardap, Khomas and Karas are considered to support incidence of <1 person per 10,000 and are therefore almost malaria free [2].

Between 2004 and 2009, about USD 12.2 million were committed to malaria control in Namibia, of which approximately 10.5 million was provided by the Global Fund to Fight AIDS, Tuberculosis and Malaria (GFATM) [25,26]. This has led to significant increase in the coverage of malaria interventions in the last decade [26]. In 2000, only 6.7% of children under the age of five years slept under a bed net and by 2009 this had increased to 34% [27]. Namibia has also achieved substantial economic progress with average annual GDP growth of about 5% from 2003 to 2010 and is now ranked by the World Bank as an upper middle income country [28]. Coincident with this economic growth has been significant increases in the rate of urbanisation from 28% at independence to almost 40% by 2010 [29]. The combination of control, economic growth and urbanisation are likely to have reduced the intrinsic malaria transmission potential of the country.

In March 2009, a meeting of the Elimination Eight (E8) countries of the Southern Africa Development Community (SADC) was held in Windhoek with the aim of eliminating malaria in the four first tier countries (Botswana, Namibia, South Africa and Swaziland) by 2015 while accelerating control with the aim of eventual elimination in the four second tier countries (Angola, Mozambique, Zambia and Zimbabwe) [1]. In 2010, a third national malaria policy and strategy was launched for the period 2010–2016 aimed at achieving a malaria case incidence of less than 1 per 1000 population by 2016 in all the districts of Namibia through universal scale up of diagnosis, treatment and prevention [2,27]. Consequently, in April 2010, the Namibian government launched a malaria elimination campaign to move the country to pre-elimination/elimination in the next five to ten years [6].

### Assembly of *P. falciparum* infection prevalence data

#### Historical malaria prevalence data (1967–1992)

In 2011 village-level data were assembled from monthly and annual reports of the parasitology department at the National Institute of Tropical Diseases (NITD) at Tzaneen, South Africa [12]. Malariologists at Tzaneen supported the routine mass blood survey examinations that accompanied malaria control in the northern territories in Namibia from the 1960s to the early 1990s across Ovambo (Ohangwena, Oshikoto, Omusati ans Oshana), Kavango, Caprivi, Bushmanland (Otjozondjupa), Hereroland (Omaheke) and Damaraland (Kunene and parts of Erongo). Thick and thin Giemsa stained blood smears were taken from individuals transported to the NITD and examined using light microscopy by expert microscopists before returning results to Namibia to support annual control planning. Majority of these surveys were undertaken among a sample of all ages in the months of March to June which is coincident with the main transmission season. These active mass blood surveys were suspended after 1992. Information on village name, month and year of the survey, numbers of people examined, numbers positive for *P. falciparum* and the age range of the surveyed community were extracted. The longitude and latitude of all survey locations were subsequently identified using a variety of digital place name databases, gazetteers and a settlement database mapped using Global Positioning Systems (GPS) receivers.

#### Contemporary malaria prevalence data (2009)

The only other national malariometric survey undertaken since 1992 was the Malaria Indicator (MIS) conducted in April 2009 [27]. This survey was designed using a two-stage probability sample, allowing for precision between urban and rural areas and three malaria risk strata (malaria absent, epidemic prone and endemic) in the nine northern regions of Namibia. Primary sampling units (clusters) were identified within a constituency, district and region. Finger prick blood samples were taken from every resident child below the age of five years whose parents or guardians provided informed consent within every cluster. In every fourth household all respondents were asked to provide a finger prick blood sample for malaria parasitology. A Rapid Diagnostic Test (RDT) (Paracheck *Pf*®, Orchid Bio-Medical Systems, Goa, India), was used to record infection at the time of the survey. Thick and thin blood smears were also made for subsequent detailed parasitology however the quality of slide preparation, staining and storage limited detailed examination of infection from the field slides and only RDT results were used in this study. Survey data were entered directly in the field using Personal Digital Assistants and transferred to STATA (Statacorp Inc., version 8) and data on age, household location and RDT results were extracted for analysis.

#### Modelling spatial receptive and current P. falciparum risks

Bayesian model-based geostatistical (MBG) methods were used to predict probable receptive and current malaria risk in Namibia. These models allow the use of the properties of the data (age, sample size, temporal and spatial structure) and carefully selected climate and ecological covariates to predict risks at un-sampled locations.

A suite of ecological and climatic covariates of malaria transmission likely to improve the precision of modelled predictions of malaria risk were assembled including urbanisation surface for 2010 [12,30], a temperature suitability index (TSI) that relates to the temporal probability of sporozoite development in cohorts of dominant vectors based on daily ambient temperatures [31], measures of water availability for larval development based on remotely sensed enhanced vegetation indices (EVI), interpolated measures of mean annual precipitation [32], and proximity to main water features [33]. TSI, EVI and precipitation were generated from long-term annual average temperature, vegetation, precipitation surfaces respectively and represent estimates of an average year. The values of these covariates were extracted to each survey location using ArcGIS 10 *Spatial Analyst* (ESRI Inc. NY, USA) tool. A total-sets analysis based on a generalized linear regression model and implemented in *bestglm* package in R [34,35] was then used to select those covariates that were most predictive of *P. falciparum* prevalence. The best combination of covariates, which was those with the lowest value of the Bayesian Information Criteria (BIC) statistic, [36] was selected for the prediction of malaria risk.

A Bayesian model-based geostatistical (MBG) framework was used to produce continuous maps of *P. falciparum* prevalence at 5 × 5 km spatial resolution. The model approach used with the historical mass blood surveys of 1967–1992 is described elsewhere [12]. A similar approach, but without the temporal dimension, was used to model the data from the MIS of 2009. Briefly, the model assumed that individuals examined for *P. falciparum* in each survey location were positive with a probability that was the product of a continuous function of the time (for the 1967–1992 data) and location of the survey and a factor that was determined by the age range of the individuals who were examined. A Gaussian random field [37] was used to model the continuous functions of time and space while the age-standardisation factors were modelled using a Bayesian version of the procedure described in [38] to provide predictions within a standard age range 2–10 years (*Pf*PR_2-10_). Bayesian inference was implemented using the Markov chain Monte Carlo algorithm. Each survey was referenced temporally using the mid-point (in decimal years) between the recorded start and end months. For each grid location samples of the annual mean of the full posterior distribution of *Pf*PR_2-10_ for the years 1969, 1974, 1979, 1984, 1989 were generated. These annual mean *Pf*PR_2-10_ maps were generated as part of previous work that described the relationship between control, environmental factors, and changing infection rates in Namibia [12].

To measure receptivity the highest value of the predicted mean annual *Pf*PR_2-10_ value at each 5 × 5 km grid location for the years 1969, 1974, 1979, 1984 and 1989 were computed. These were then combined to generate a single map of maximum mean *Pf*PR_2-10_ at each grid location. Both the maximum mean and the 2009 predictions were used to generate the endemicity classes: (*Pf*PR_2-10_ <1% (low stable endemic control); *Pf*PR2_−10_ 1- < 5% (hypoendemic 1); *Pf*PR_2-10_ 5- < 10% (hypoendemic 2); *Pf*PR_2-10_ 10- ≤ 50% (mesoendemic); *Pf*PR_2-10_ >50% (hyper- and holo-endemic).

Model accuracies were estimated by computing the linear correlation, the mean prediction error (MPE) and mean absolute prediction error (MAPE) of the observations and predictions to a 10% hold-out dataset. MPE and MAPE are measures of overall model bias and accuracy respectively. The hold-out set was selected separately for 1967–1992 and 2009 data series. The hold-out set was selected using a spatially and temporally declustered algorithm [39] which defined Thiessen polygons around each survey location. Each data point had a probability of selection proportional to the area of its Thiessen polygon so that data located in densely surveyed regions had a lower probability of selection than those in sparsely surveyed regions setting a high threshold for model performance. The Bayesian spatio-temporal geo-statistical model was then implemented in full using the remaining 90% of data and predictions were made to the 10% hold-out.

#### Mapping populations at risk and computing population adjusted PfPR_2-10_

Combinations of remotely sensed, re-classified landcover data [30,40,41], administrative boundaries, towns and settlements point locations, health facilities and schools locations, transport networks and the 2001 population count census data for 4072 enumeration areas (average spatial resolution of 14.3 km^2^) were used to define per land cover class population densities at 100 × 100 m resolutions [28]. The population count data were adjusted forward to the estimated 2010 levels using separate UN urban and rural growth rates for Namibia [29].

The 2010 projected population surface was used to extract population counts by endemicity class and compute population adjusted *Pf*PR_2-10_ (PA*Pf*PR_2-10_) by health district for both the receptive and contemporary (2009) risk maps. PA*Pf*PR_2-10_ for each district was computed by first extracting the population count for each 5 × 5 km grid to which *Pf*PR_2-10_ predictions were made. The *Pf*PR_2-10_ (in proportions) then multiplied by the population count at each 5 × 5 grid location to compute the number of people likely to be positive at that location. The estimated positive cases and the total population counts were then summarised for each health district and the district-specific PA*Pf*PR_2-10_ was computed. Population extractions and multiplication of surfaces was undertaken using the *Spatial Analyst* tool in ArcGIS 10 (ESRI Inc. USA).

## Results

The empirical data assembled comprised of 3,260 geo-coded community *Pf*PR surveys covering 230,178 people between 1967 and 1992 [12] and 120 community surveys covering 4,572 people during the 2009 MIS. For both the historical and contemporary data series the covariates that were selected in the final best-fit model as predictors of *Pf*PR_2-10_ included EVI, precipitation and urbanisation.

**Figure 2:**
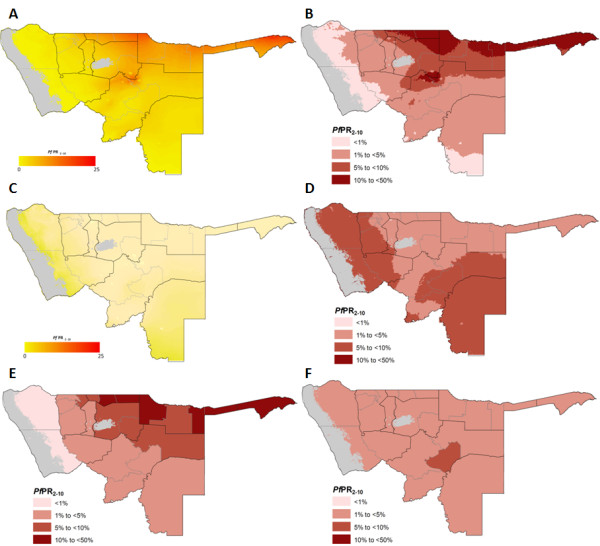
**Maps of northern Namibia showing: A) The continuous maximum mean (receptive) *Pf*PR**_**2-10 **_**at 5 x 5 km location from the posterior mean distribution of *Pf*PR**_**2-10 **_**for the years 1969, 1974, 1979, 1984 and 1989; B) endemicity classes constructed from the *Pf*PR**_**2-10 **_**continuous receptive risk map; C) the continuous posterior mean distribution of *Pf*PR**_**2-10 **_**for 2009; D) endemicity classes constructed from the *Pf*PR**_**2-10 **_**continuous 2009 risk map; E) PA*Pf*PR**_**2-10 **_**receptive risks by health district; F) PA*Pf*PR**_**2-10 **_**risks for 2009 by health district.**

The receptive (maximum mean) *Pf*PR_2-10_ continuous and endemicity risk maps are shown in Figure 2A and B respectively. These maps show that majority of the northern regions have receptive risks of > = 5% *Pf*PR_2-10_ with the whole of Caprivi, most of Ohangwena, large parts of Kavango and a small pocket in the neighbouring Otjozondjupa exposed to risks of >10% to an upper predicted limit of 25% *Pf*PR_2-10_. Based on the predictions to a 10% holdout dataset, the linear correlation of the predicted and observed *Pf*PR_2-10_ was 0.61. The MPE and MAPE were also 1.6% and 7.5% respectively. In contrast, the contemporary 2009 map indicates that most of the historical malarious northern regions are now exposed to risks of <5% *Pf*PR_2-10_ (Figure 2C & D). However, a minor rise in risk was predicted in the historically very low transmission areas of Omaheke and Kunene regions which now support *Pf*PR_2-10_ risks of 5% to 10%. The model MAE was 3.3% while the MAPE of 9.7% showing accuracies that are lower than those of the historical data model.

Approximately 0.8 million (57%) of the population of northern Namibia reside in areas where receptive risks are likely to be ≥5% *Pf*PR_2-10_ (Table 1). By 2009 only 15% (0.2 million) of the population of these regions were exposed to risks of the same endemicity class. Relative to their receptive endemicities, the regions of Caprivi, Kavango, Ohangwena, Omusati, Oshana and Oshikoto all had more than 80% of their populations currently residing in a lower contemporary endemicity class, mainly between 1% and 5% *Pf*PR_2-10_ (Table 1 and Figure 2E). Estimates at the health district level show overall current PA*Pf*PR_2-10_ were <5% except in Okarara district in Otjozondjupa region (Figure 2F). However, in terms of receptive risks 12/23 districts had PA*Pf*PR_2-10_ ranging from 5% to 18% and were located mainly in Caprivi, Kavango, Ohangwena, Omusati, Oshikoto and also accounted for 57% of the population of the northern regions (Table 1 and Figure 2E).

**Table 1:** **The 2010 population count at different *Pf*PR**_**2-10 **_**receptive (maximum mean for 1969–1989) and current (2009) endemicities in the nine northern regions of Namibia**

	**<1% *Pf*PR**_**2-10**_		**1% to <5% *Pf*PR**_**2-10**_		**5% to 10% *Pf*PR**_**2-10**_		**>10% to 50% *Pf*PR**_**2-10**_		**Total 2010 population**
**Region**	**Receptive**	**Current**	**Receptive**	**Current**	**Receptive**	**Current**	**Receptive**	**Current**	
Caprivi	0	0	0	82,099	4,310	0	77,789	0	82,099
Kavango	0	0	0	157,351	42,869	0	114,481	0	157,351
Kunene	33,950	0	30,885	11,297	1,250	54,231	557	0	66,084
Ohangwena	0	4,811	0	242,556	148,259	0	99,109	0	247,368
Omaheke	64,978	0	30,471	49,795	1	45,656	0	0	95,450
Omusati	0	0	156,743	213,860	99,841	44,569	1,844	0	258,429
Oshana	0	2,046	82,300	144,266	64,116	104	0	0	146,416
Oshikoto	0	0	36,867	179,083	135,759	0	6,456	0	179,083
Otjozondjupa	26,025	0	122,117	100,448	14,885	65,268	2,689	0	165,716
**Total**	**124,953**	**6,857**	**459,383**	**1,180,754**	**511,291**	**209,827**	**302,926**	**0**	**1,397,995**

## Discussion

In Namibia the highest population density is coincidental with the highest receptive risks of malaria transmission along the border with Angola and Zambia (Figure 2A, B and E). Relative to the receptive risks, current risk estimates show that over 85% of the approximately 1.4 million people of northern Namibia have transitioned to endemicity levels that are at least one class lower and PA*Pf*PR_2-10_ by 2009 were largely <5% (Figure 2, Tables 1 &2). Moderate increases in risk were predicted in Kunene and Omaheke regions in 2009. It is possible that this rise is due to the emergence of focal hotspots of transmission as in-migration into these areas increased, or that infections were observed in individuals who frequently travel to the higher risk northern border areas or because of model uncertainties as a result of the few MIS survey clusters. Nonetheless, of the 23 health districts in northern Namibia, 12 have receptive risks of between 5% and 18% PA*Pf*PR_2-10_ and are mainly in Caprivi, Kavango and the regions of the Ovambo (Figure 2 & Table 2). From a programmatic perspective, their relative high receptivity and proximity to the border with Angola and Zambia make these districts at highest risk of the rebound of transmission due to importation and should be priority for sustained control.

**Table 2:** **The 2010 population count at different population adjusted *Pf*PR**_**2-10 **_**(PA *Pf *PR**_**2-10**_**) receptive (maximum mean for 1969–1989) and current (2009) endemicities in the health districts of the northern regions of Namibia**

**Region**	**Health district**	**Population 2010**	**Receptive PA *Pf *PR**_**2-10**_	**Current (2009) PA *Pf *PR**_**2-10**_
Caprivi	Katima	82,099	18.1	3.7
Ohangwena	Kongo	18,309	14.5	4.6
Kavango	Nyangana	16,302	13.6	4.3
Kavango	Andara	32,626	13.4	3.7
Kavango	Nankudu	32,828	12.7	4.4
Ohangwena	Eenhana	61,277	10.2	4.2
Kavango	Rundu	75,595	9.6	3.6
Ohangwena	Engela	167,782	9.4	3.7
Omusati	Oshikuku	101,945	6.8	4.2
Oshikoto	Tsumeb	20,398	6.3	2.3
Oshikoto	Onandjokwe	158,685	6.0	4.3
Otjozondjupa	Grootfontein	34,172	5.3	3.7
Oshana	Oshakati	146,416	4.4	2.8
Omusati	Outapi	77,043	4.1	4.9
Omusati	Tsandi	45,138	3.9	4.9
Omusati	Okahao	34,304	3.5	4.5
Otjozondjupa	Okakarara	16,990	2.7	5.4
Otjozondjupa	Otjiwarongo	76,081	2.7	3.6
Kunene	Outjo	17,619	1.9	4.9
Otjozondjupa	Okahandja	38,472	1.4	2.7
Omaheke	Gobabis	95,450	1.2	4.3
Kunene	Opuwo	30,545	0.9	3.4
Kunene	Khorixas	17,921	0.4	3.1
**Total**		**1,397,995**	**6.8**	**3.9**

Health districts are sorted by highest to lowest receptive PA*Pf*PR_2-10_.

Frequent population movement across this border during the late 1970s and 1980s as a consequence of the war for independence was thought to have contributed to the challenges of malaria control in this area [8,10,12]. Familial links, commerce, border trade zone agreements and access to health care between Angola and Namibia continues to result in large population movement [42,43]. For example at Oshikango, one of the busiest official entry points, the annual number of foreign citizens crossing into Namibia almost doubled from 144,000 in 1999 to 267,500 in 2003 and number of Namibians crossing to Angola almost doubled from approximately 26,000 (1999) to over 61,000 (2003); between 1999 and 2003 there were over 1.3 million arrivals from Angola [41]. Mathematically it is important to distinguish the four mechanisms by which malaria parasites can be imported into an area: residents of the area can become infected while travelling elsewhere before returning home; visitors from another area can import infections and return home after transmitting the parasite; immigrants infected elsewhere can move permanently into the region; or infected mosquitoes travel into the area [16,44]. Given the distances within the flight range of *An. arabiensis*, mobility, migration and receptivity across certain parts of northern Namibia this will continue to pose a threat to elimination ambitions for Namibia by 2020.

Receptivity is difficult to quantity and here we have used a space-time MBG framework to provide predictions of risk every five years from 1969 to 1989 and assumed the maximum prediction per 5 × 5 km grid to represent the maximal extent of predicted risk. The data with which the model was developed were collected at a time when vector control and anti-malaria drug use were deployed extensively, especially in Ovambo, Kavango and Caprivi regions and therefore our estimates of receptive risk, while higher than current risk, is not a measure of intrinsic transmission potential in the absence of control. Insufficient data exist pre-1969 to provide a measure of transmission before control in Namibia. In addition, there are no *Pf*PR data from 1993 to 2004, a period covering the reported epidemics of 1996, 1997, 2000, 2001 and 2004 [2]. However, recent analysis has shown that between 1969 and 1989 malaria prevalence declined marginally and finally rose again by 1989 to levels similar to 1969 [12] and therefore, when summed as maximal prediction, likely to represent a reasoned, spatially relative, measure of the worst case-scenario of *P. falciparum* transmission intensity in this area [45]. This must be interpreted against a background of significant economic expansion [28] and increased urbanisation that are likely to have transformed the intrinsic risk of transmission in some areas, to the extent that even if control were interrupted, rebound to historical risks of transmission are unlikely. This must however be balanced with the fact that these economic expansion and increased urbanisation have been concentrated around the central and southern parts of the country where the ecology naturally supports very low malaria transmission while in the northern malarious areas, the majority of the population is still rural and have the highest rates of poverty in the Namibia [3].

For time-dependent modifiers of malaria transmission such as climatic, ecological, economic and control factors to be fully adjusted for in the predictive model, they have to available both for the sampled locations (survey clusters) and for the entire predictive surface. Such data are not available in this form thereby limiting their utility in malaria risk mapping. Most of the available environmental data are derived from remotely sensed satellite sources and are only available at least monthly at high spatial resolution for the last decade. For this reason, as is the common practice in malaria risk mapping, we have used synoptic (long term average) covariate data which do not correspond to a given year but are representative of an average year. As for urbanisation, we have used a surface of 2010 due to lack of historical urbanization maps and the effect of this is likely to be a slight underestimation of historical risk.

There is also less certainty about current parasite prevalence, due largely to the limited distribution of data from the MIS of 2009, which was population-weighted and powered to examine prevention coverage rather than spatially weighted malaria infection prevalence. Additionally, the MIS was based on Paracheck-*Pf*® RDT results which have a documented false positive rate [46,47], thus over-predicting risks. The intrinsic sampling and the subsequent spatial modelling uncertainty of the MIS data and the reliance on RDT results should be addressed to provide a more robust estimate of the current distribution of infection risk in northern Namibia. However, the prediction of risk using these data provide some useful indications of how low current infection risks are and where the highest risks would be predicted given the spatial properties of these data.

Vulnerability to cross-border importation of risk from the higher transmission countries such as Angola and to a lesser extent Zambia, Zimbabwe and Botswana has been recognised as a major threat to elimination by 2020 in Namibia. It is for this reason that the Government of Namibia has signed the Trans-Zambezi Malaria control in 2006 in partnership with Angola, Zambia, Zimbabwe and Botswana to undertake cross-border malaria control along the Zambezi River [14]. On the Namibian side this initiative covered the regions of Caprivi and Kavango. In 2011, Namibia also signed Trans-Kunene Malaria Control Initiative with Angola to substantially scale-up control on both sides of the districts along the River Kunene and on the Namibian side included the border districts in Kunene, Ohangwena and Omusati regions [13]. The regions targeted in both cross-border initiatives are also shown in the receptive *Pf*PR_2-10_ risk map (Figure 2E) as largely those at the greatest risks of re-introduction of transmission in the event of an importation of infection.

## Conclusions

To fully define the risks posed by human population movement to areas where risks are now low but have a high malariogenic potential a more detailed enquiry is necessary on the patterns and volumes of mobility and migration. Techniques and approaches to monitoring human population movement are evolving rapidly to support a broad range of infectious diseases [48] and malaria specifically [16,49], most notably in relation to the use of anonymysed cell phone records to map movement between areas of high and low malaria risk [50]. Defining receptive risk remains an important component and demands the use of pre-intervention data and careful selection of a most parsimonious period likely to represent a point of rebound should malaria transmission re-establish. We would however argue that this is equally important in all areas where malaria burdens are declining, not only those countries or areas poised for elimination. In Somalia for example the intensity of transmission today is likely to be a direct result of recent droughts and maps of current risk do not represent the true risk necessary to design control [44]. Should investment in malaria control across Africa be interrupted we increasingly need to be able to articulate the risk of rebound [51]. The receptive risk map for malaria in Namibia developed here provides the necessary epidemiological evidence to guide the control activities envisaged under both the Trans-Kunene and Trans-Zambezi initiatives.

## Competing interests

The authors declare that they have no competing interests.

## Authors’ contribution

AMN was responsible for overall scientific management, study design, data cleaning, analysis, interpretation, drafting and production of the final manuscript. RNK, BN and SK contributed to the interpretation of results and drafting of the manuscript. VAA was responsible for data cleaning, geo-coding, analysis and contributed to the final manuscript. RWS provided overall scientific guidance and contributed to the data assembly, analysis, interpretation and preparation of the final manuscript. All authors read and approved the final manuscript.

## Pre-publication history

The pre-publication history for this paper can be accessed here:

http://www.biomedcentral.com/1471-2334/13/184/prepub
